# Prevalence, increase and predictors of family violence during the COVID-19 pandemic, using modern machine learning approaches

**DOI:** 10.3389/fpsyt.2022.883294

**Published:** 2022-08-11

**Authors:** Kristina Todorovic, Erin O’Leary, Kaitlin P. Ward, Pratyush P. Devarasetty, Shawna J. Lee, Michele Knox, Elissar Andari

**Affiliations:** ^1^Department of Psychology, University of Toledo, Toledo, OH, United States; ^2^Department of Psychiatry, College of Medicine and Life Sciences, University of Toledo, Toledo, OH, United States; ^3^School of Social Work, University of Michigan, Ann Arbor, MI, United States; ^4^College of Medicine and Life Sciences, University of Toledo, Toledo, OH, United States

**Keywords:** COVID-19, child maltreatment, intimate partner violence, anxiety, depression, machine learning, random forest

## Abstract

**Background:**

We are facing an ongoing pandemic of coronavirus disease 2019 (COVID-19), which is causing detrimental effects on mental health, including disturbing consequences on child maltreatment and intimate partner violence.

**Methods:**

We sought to identify predictors of child maltreatment and intimate partner violence from 380 participants (mean age 36.67 ± 10.61, 63.2% male; Time 3: June 2020) using modern machine learning analysis (random forest and SHAP values). We predicted that COVID-related factors (such as days in lockdown), parents’ psychological distress during the pandemic (anxiety, depression), their personality traits, and their intimate partner relationship will be key contributors to child maltreatment. We also examined if there is an increase in family violence during the pandemic by using an additional cohort at two time points (Time 1: March 2020, *N* = 434; mean age 35.67 ± 9.85, 41.69% male; and Time 2: April 2020, *N* = 515; mean age 35.3 ± 9.5, 34.33%).

**Results:**

Feature importance analysis revealed that parents’ affective empathy, psychological well-being, outdoor activities with children as well as a reduction in physical fights between partners are strong predictors of a reduced risk of child maltreatment. We also found a significant increase in physical punishment (Time 3: 66.26%) toward children, as well as in physical (Time 3: 36.24%) and verbal fights (Time 3: 41.08%) among partners between different times.

**Conclusion:**

Using modernized predictive algorithms, we present a spectrum of features that can have influential weight on prediction of child maltreatment. Increasing awareness about family violence consequences and promoting parenting programs centered around mental health are imperative.

## Introduction

COVID-19 is an infectious disease that is caused by severe acute respiratory syndrome coronavirus 2 (SARS-CoV-2) and is primarily spread *via* social interactions and face-to-face exchanges. Prior to the COVID-19 pandemic, family violence was a major public health issue, with 678,000 children as victims of maltreatment in 2018 and one in every four females and one in every nine males as victims of intimate partner violence (IPV) (1). Given this profound prevalence and the current pandemic, the risk of child maltreatment and IPV may be at an elevated risk.

Several factors, such as economic hardship, uncertainty for the future, fear of getting infected, working from home, homeschooling, less support from other caregivers, and social isolation can lead to an increased level of stress and have a negative impact on mental health (2). The COVID-19 pandemic has been associated with psychological difficulties such as depression and anxiety (3–5). Depression and anxiety are associated with an increased risk of child maltreatment and neglect (6). In addition to psychological difficulties, the isolation caused by the pandemic, associated shelter-at-home directives, and school and service closures significantly inhibit our society’s abilities to identify and report family violence. The lack or very limited contact between children and many professionals during the pandemic provides significantly less opportunity for child maltreatment identification and reporting. Parents are having significantly more time with children and less respite and support from other, typical caregivers such as childcare workers, causing increased stress on parents. Many parents are experiencing frustration when trying to work from home while caring for children, managing children’s behaviors, and maintaining work privacy. Children and teens themselves are also more stressed due to worries about the virus, as well as needed adaptations and changes to education. These factors are likely to significantly increase negative parent-child interactions. This, coupled with higher parent stress, may significantly increase the risk of corporal punishment, abuse, and neglect. This is in keeping with evidence from previous pandemics that found parents facing health challenges were more likely to have harsher discipline strategies (7). Also, more recent work has shown that job loss during the COVID-19 pandemic has been found to be a significant risk factor for child maltreatment (8) and IPV (9). Some alarming numbers of child maltreatment during the COVID-19 pandemic have been reported with sixty-one percent of parents using verbal aggression and 1 in 5 parents using physical punishment as their discipline method (3). Therefore, psychological distress can negatively impact parenting practices and may increase the risk of negative parent-child interactions, corporal punishment, neglect, and IPV (10). Protective factors are also important to consider when addressing family violence. Parental empathy involves the ability to understand children’s feelings and intentions, as well as being able to provide comfort and show sensitivity and flexibility to children’s needs. Research has demonstrated poor parental empathy is associated with physical abuse potential and likelihood to punish (11).

The present study investigates mental health consequences of the pandemic, with an emphasis on indicators of child maltreatment and IPV. Specifically, we examined whether the pandemic has a significant impact on verbal aggression and physical punishment toward children and on violence between partners. We first adopted state-of-the-art machine learning approaches to determine the relative contribution of independent factors related to the pandemic among other factors related to mental health and personality traits of parents, to predict verbal aggression and physical punishment toward children, and IPV at Time 3. We used random forest analysis to capture non-linear decision boundaries and nuanced interactions amongst features. We expect COVID-19 related items will be predictive of increased violence, and psychological well-being and emotional empathy will be strong predictors of lower incidence of family violence. Second, we aim to study the potential increase in family violence during the pandemic by comparing participants’ reports of child maltreatment and IPV between different time points of the pandemic, (Time 1: March 2020, Time 2: April 2020, and Time 3: June 2020). We predict that there will be an increase of verbal aggression and physical punishment toward children and IPV over time.

## Materials and methods

### Participants

#### Time 1 and time 2

*For Time 1*, a total of 434 parents or/and participants in a relationship (mean age 35.67 ± 9.85, 41.69% male); and *For Time 2*, a total of 515 parents or/and participants in a relationship (mean age 35.3 ± 9.5, 34.33% male) were included in the analysis. These participants were recruited via online surveys that were administered through Prolific. Time 1 was collected in March 2020 and Time 2 was collected in April 2020. Participants were United States citizens and were age 18 or older. On average for both times, 74.33% were Caucasian, 9.37% were African American, 8.52% were Hispanic, 3.3% were Asian/Native Hawaiian/Other Pacific Islander, 3.19% were biracial, 0.85% were other and 0.43% were American Indian/Alaskan Native. 37.72% were male participants, 44.28% are from the South state region, 20% from the Midwest, 20% from the West, and 15.72% from Northeast. The distribution of income ranged between $10,000 and more than $90,000. For education level, 25.85% had some college, 35.80% had bachelor’s degree, 10.91% had high school, 10.8% had an associate degree and 15.68% had a Master’s, professional, doctorate, and 0.95% no high school. See Table 1.

**TABLE 1:** Descriptive statistics of measures in the present study.

Measures		*n*	Percent (%)	Min	Max	*M*	*SD*
IRI-EC				0	28	17.72	5.32
PHQ-8				0	21	9.75	6.89
GAD-7				0	19	8.28	5.80
DASS-21				0	57	20.37	15.83
**COVID-19 Related Personality and Emotional States**							
	Extraverted			1	7	4.11	1.75
	Aggressive			1	7	3.85	1.81
	Outspoken			1	7	4.39	1.51
	Isolated			1	7	4.98	1.49
	Like people			1	7	4.03	1.75
	Frustrated			1	7	4.49	1.66
	Chatting			1	7	3.98	1.72
	Empathic			1	7	4.83	1.37
	Stressed			1	7	4.37	1.57
	Emotions			1	7	4.79	1.30
	Anxious/Worry			1	7	4.44	1.68
	Scared			1	7	4.13	1.78
**Parent-Child Conflict Tactics Scales^1^**							
	Verbal aggression toward child in past 2 weeks	191	76.71				
	Physical punishment toward child in past 2 weeks	165	66.26				
	Shouted, yelled, screamed at child(ren)	186	74.7				
	Called child(ren) dumb or lazy or some other name like that	157	63.31				
	Spanked child(ren)	160	64.52				
	Slapped child(ren) on the hand, arm, or leg	153	61.94				
**Romantic relationships^2^**							
Agree Strongly Agree	My spouse or partner and I have had disagreements related to the Coronavirus/COVID-19 global health crisis	131	43.96	1	5	2.91	1.30
	My spouse or partner and I have had more disagreements than usual	111	37.25	1	5	2.84	1.38
	My spouse or partner and I have had more verbal fights than usual	122	41.08	1	5	2.86	1.35
	My spouse or partner and I have had more physical fights than usual	108	36.24	1	5	2.71	1.44
How tired are you from taking care of your children AND trying to work from home?^1^				1	7	5.08	1.89
How much time (in hours) does your child spend in front of a screen per day?^1^						3.58	1.72
How significantly has your life been disrupted by the Coronavirus/COVID-19 global health crisis?				1	5	3.47	.99
**What activities are your children doing these days?^1,3^**							
	Physical games	143	57.2				
	Electronic games	124	49.6				
	Reading	161	64.4				
	Puzzles	118	47.2				
	Writing skills	92	36.8				
	Outdoor activities	68	27.2				
# of “yes” responses to using the resources provided in the debriefing form		219	57.6				

^1^Only participants who indicated having children (n = 250) were given survey items related to their children. ^2^Only participants who reported being in a romantic relationship (n = 298) were asked these items. For the verbal fights among partners item, one participant left this item blank, thus, this item is based off 297 participants. ^3^Participants were able to select multiple activities that their child took part in, thus, in our sample of the 250 participants with children the selection of activities ranged from 68 (outdoor activities) to 161 (reading) (Time 3 dataset).

#### Time 3

A total of 380 adults from 43 states in the United States were recruited from Amazon’s Mechanical Turk (Mturk) from June 5, 2020 to June 14, 2020 (Time 3). Mturk is a crowdsourcing website that allows researchers to conduct research including participant recruitment, data collection, and compensating participants. Mturk has been shown to obtain high-quality data from participants and includes a demographically diverse sample (12). The inclusion criteria for the present study included (1) United States resident, (2) at least 95% approval rating as an MTurk worker (e.g., workers with a high completion and quality rating), (3) valid responses on questionnaire. Participants (36.3% female, 63.2% male, 0.3% prefer not to answer) ranged in age from 20 to 73 (*M* = 36.67, *SD* = 10.61).

Most participants identified as White/Caucasian (78.7%), followed by Black/African American/African Diaspora (12.1%), Asian/Native Hawaiian/Other Pacific Islander (6.3%), American Indian/Alaskan Native (1.3%), and Other/Prefer not to answer (1.6%). More than half of participants reported living in an urban neighborhood (53.2%), followed by suburban (28.4%) and rural (18.4%). 78.4% of participants reported being in a romantic relationship and 65.8% of participants reported having 1 to 6 children. Our sample represented all regions of the United States: 13.4% Northeast, 16.3% Midwest, 39.2% South, and 30.5% West.

We also collected additional demographics between May 24, 2021 to May 27, 2021 from 106 of the original participants from Time 3. Among parents or/and participants in a relationship (*n* = 68), around 32.35% have an income that ranges between $40–$50k, 33.82% have an income that ranges between $50–$70k, 13.24% have an income that ranges between $30–$40k, 8.82% that ranges between $70–$90k, and 8.82% that ranges between $20–30k. 60.29% have a bachelor’s degree, 20.59% have some college, 11.76% have a master’s, professional, doctorate, and 4.41% have an associate degree, and 2.94% are in high school.

### Procedures

#### Time 1 and time 2

The average completion time for Time 1 was 33 min and 32 min for Time 2. For Time 2, they were recontacted via the Prolific website. Data from these time points was provided as anonymous therefore, the data was deemed exempt from the first authors university’s Institutional Review Board.

#### Time 3

All procedures received approval from the first authors university’s Institutional Review Board. Participants completed a 1-hour survey exploring people’s social and emotional experiences during the COVID-19 pandemic. To ensure that participants provided valid responses on the questionnaires we included 10 explicit request attention check items (e.g., If you are still paying attention, please select “once”). On the consent form, participants were informed that we placed attention check questions throughout the survey and participants had to answer these questions correctly to receive compensation. Participants had to respond correctly to the 10 attention check questions to be included in our sample. Data were collected in blocks of 10 participants then examined by the first author. Participants whose data were believed to be invalid were removed from the study (*n* = 20 of 400 participants).

### Measures

#### Dependent variables

##### Child maltreatment

Items from the Parent–Child Conflict Tactics Scales were used to assess risk for verbal aggression and physical aggression toward children at all time-points (13). We modified the response scale to ask participants how often behaviors occurred “in the past two weeks” on a 0 (“never”) to 6 (“every day”) scale. We computed dichotomous variables for verbal aggression and physical aggression to indicate no verbal aggression/physical aggression or one or more instances of verbal aggression/physical aggression in the past two weeks. Verbal aggression was measured with two items (i.e., “Shouted, yelled, or screamed at child(ren)” and “Called child(ren) dumb or lazy or some other name like that”). Physical aggression was measured with two items (i.e., “Spanked child(ren)” and “Slapped child(ren) on the hand, arm, or leg”). Internal consistency was excellent (Time 3 α = 0.94).

##### Intimate partner relationships

Information about intimate partner relationships was measured with 6-items about their relationships in the past 2 weeks at all time-points (3). For each item, participants choose one response ranging from 1 (“strongly disagree”) to 5 (“strongly agree”). Sample items include, “My spouse or partner and I have had more verbal fights than usual,” and “My spouse or partner and I have had more physical fights than usual”).

#### Predictive features

Several questions were asked that are related to the following domains: (1) *Empathic concern* (Interpersonal Reactivity Index, IRI) (14). At Time 3, empathy was measured with the IRI which is a 28-item scale measuring perspective taking, fantasy, empathic concern, and personal distress. Participants in the present study were only given seven items from the empathic concern subscale. All items were rated on a 0 (“does not describe me well”) to 4 (“describes me very well”) scale. Items were reversed scored as needed. Internal consistency was acceptable (Time 3 α = 0.74); (2) *Psychological distress over the last two weeks* (depression evaluated with the 8-item Patient Health Questionnaire (PHQ-8); (15), anxiety evaluated with the Generalized Anxiety Disorder Assessment (GAD-7); (16), and 21-items Depression Anxiety Stress Scale (DASS-21); (17) to ensure intra-individual consistency for depression and anxiety) For all time points, severity of depressive symptoms was measured with the 8-item PHQ-8. The PHQ-8 asked participants “Over the last two weeks, how often have you been bothered by any of the following problems?” Participants rated each statement on a 4-point scale ranging from 0 (“not at all”) to 3 (“nearly every day”). A sum of the 8-items was created for data analyses in the present study. Internal consistency in the present sample was excellent (Time 3 α = 0.93). For all time points, anxiety was measured with the Generalized Anxiety Disorder Assessment (GAD-7). The GAD-7 is a 7-item scale assessing severity of generalized anxiety disorder. Participants are asked to rate the severity of symptoms over the past two weeks on a 4-point scale ranging from 0 (“not at all”) to 3 (“nearly every day”). Internal consistency was excellent in the present sample (Time 3 α = 0.91). In addition to separate measure of depression and anxiety, we also included the 21-item Depression Anxiety Stress Scales at Time 3 and 4 (DASS-21) to ensure intra-individual consistency in terms of feelings of depression and anxiety. Participants were given a series of statements and asked to rate each item on a 4-point scale ranging from 0 (“did not apply to me at all”) to 3 (“applied to me most of the time”) to indicate how much the statement applied to them over the past week. Internal consistency was excellent (Time 3 α = 0.97); (3) *COVID-19 related daily life* (such as days in lockdown, social distancing, life disruption); (4) *COVID-19 related childcare* (tired of taking care and homeschooling); (5) *COVID-19 related personality and emotional states* (aggressivity, extraversion, stress, frustration, anxiety, scared, empathy experienced during the COVID-19 pandemic). At Time 3, COVID-19 related personality and emotional states items were coded on a 7-point scale ranging from 1 (“not at all”) to 7 (“extremely”). These items measured participants COVID-19 related experiences via a 17-item measure developed for this study. Participants were asked about their personality and emotional state during the COVID-19 pandemic. The COVID-19 related personality and emotional states questions consisted different questions related to different constructs that are the following: extraversion (“How extraverted [outgoing, sociable] would you rate yourself now?”), aggression (“How aggressive would you rate yourself during the COVID-19 pandemic?”), and empathy (“How empathic would you rate yourself during the COVID-19 pandemic?”), frustrated (“How frustrated would you rate yourself during the COVID-19 pandemic?”), stressed (“How stressed would you rate yourself now?”), anxious/worry (“How often do you feel anxious or worried now?”), and scared (“How often do you feel scared now?”); (6) *Children’s activities* (such as activities outdoor, writing skills, screen time, reading, puzzles, electronic games), (7) *Need for help* (interested in requesting help) (8) *Intimate partner relationship* (COVID-19-related disagreements, disagreements in general, physical fights, and verbal fights since the start of the pandemic), and (9) *Number of children*.

### Data preprocessing

Models were constructed to predict the probability or classify instances of verbal aggression or physical punishment occurring. As such, we considered three models, an unregularized logistic regression, a regularized multiple logistic regression model, and a random forest model. There were several different types of covariates: continuous, nominal, and ordinal. Nominal variables were dummy-coded, dropping the first value. Ordinal variables were coded by centering at 0, and then performing unit steps in appropriate directions (i.e., –1, 0, 1). Missing data was handled with simple imputation.

### Statistical analysis

The unregularized logistic regression model was constructed to test for each covariate’s individual contribution, while controlling for age, gender, race, income, education, and state region. Next, we considered a regularized logistic regression model that utilized an elastic-net penalty, using scikit-learn to fit this model (18). Finally, we considered a random forest model, again using scikit-learn (19). Random forests are an ensemble supervised learning method non-linear classifier that have been trained on different subsets of the data, often perform extremely well at the cost of model interpretability. While random forests do offer insight into their model by ranking feature importance, we chose instead to use shapley additive explanations (SHAP) values. SHAP values have the advantage of guaranteeing local explanations are consistent and locally accurate, and that the global explanations are built off a series of local explanations.

Final metrics were obtained by using nested cross-validation. Nested cross-validation has been shown to be a robust technique to assess model performance and generalization (20). Instead of using K-fold Cross-Validation which produces biased performance estimates with small sample sizes, we used nested cross- validation and train approaches that can provide unbiased performance estimates regardless of sample size.

When comparing the differences between three time points (Time 1, Time 2, and Time 3), we conducted a logistic regression for the binary-response variables (physical punishment and verbal aggression toward children). In the model, age, race, gender, state region, education, income, and interaction between gender and time were included. We were interested in studying differences in gender with respect to family violence. Time was ordinally encoded. These logistic regressions models were unregularized, which allowed us to generate *p*-values and confidence intervals (CI) that correspond to the coefficients of the features. We also conducted ordinal regressions on the Likert-scale dependent variables of intimate partner relationships (physical and verbal fights). Same factors above were included in this model.

*In aim 1, to determine predictors of family violence, we used dataset of Time 3 which was our main dataset that included many independent factors. In aim 2, to determine family violence prevalence increase, we used datasets of Times 1, 2, and 3 with a focus on dependent factors and demographic information.*

### Machine learning – metrics and cross validation

Final metrics were obtained by using nested cross-validation. Nested cross-validation has been shown to be a robust technique to assess model performance and generalization. Instead of using K-fold Cross-Validation which produces biased performance estimates with small sample sizes, we used nested cross-validation and train approaches that can provide unbiased performance estimates regardless of sample size.

Briefly, (e.g.) 10-fold cross-validation involves splitting a dataset into 10 equally sized *folds*. Each fold is treated as a *validation* once, while the rest of the data is then used for training or hyper-parameter optimization, i.e., the first 10% of the data is withheld, while the remaining 90% of the data is used to train, then the next 10% of the data is withheld, and again, the remaining 90% of the data is used for training. This process continues with each fold being used as a validation set once.

In *Nested cross-validation*, an additional layer of separation between model performance metrics and hyper-parameter estimation is introduced. Like in (e.g.) 10-fold cross-validation, the dataset is split into 10 equally sized folds. However, instead of immediately using the remaining 9-folds for training, *that* dataset is further split into another (e.g.) 10-folds. Using these 10 newly created folds (that were generated from the 90% of the data being used for training) regular cross validation is performed, i.e., one (inner) fold is left out for validation, while a model is training on the remaining nine (inner) folds. This process is repeated until all (inner) folds have been used for validation. Once that process has been completed, the resulting model is then validation against the (outer) 10% of the data that has been withheld for validation. In this manner, the model is validated on against a dataset that is has never seen before. This process is repeated once for each of the (e.g.) 10 folds, resulting in a final validation metric. This nested cross-validation approach has been studied, proving to be a nearly unbiased estimate of model performance, while the regular CV approach is shown to be biased.

## Results

### Aim 1: Predictors of child maltreatment: Unregularized regressions

At Time 3, 76.4% reported one or more instances of verbal aggression toward children, 66% reported one or more instance of physical punishment toward children, 41.08% reported agreeing or strongly agreeing that they had more verbal fights with their partner than usual, and 36.24% reported agreeing or strongly agreeing that they had more physical fights with their partner than usual.

#### Verbal aggression

**FIGURE 1:**
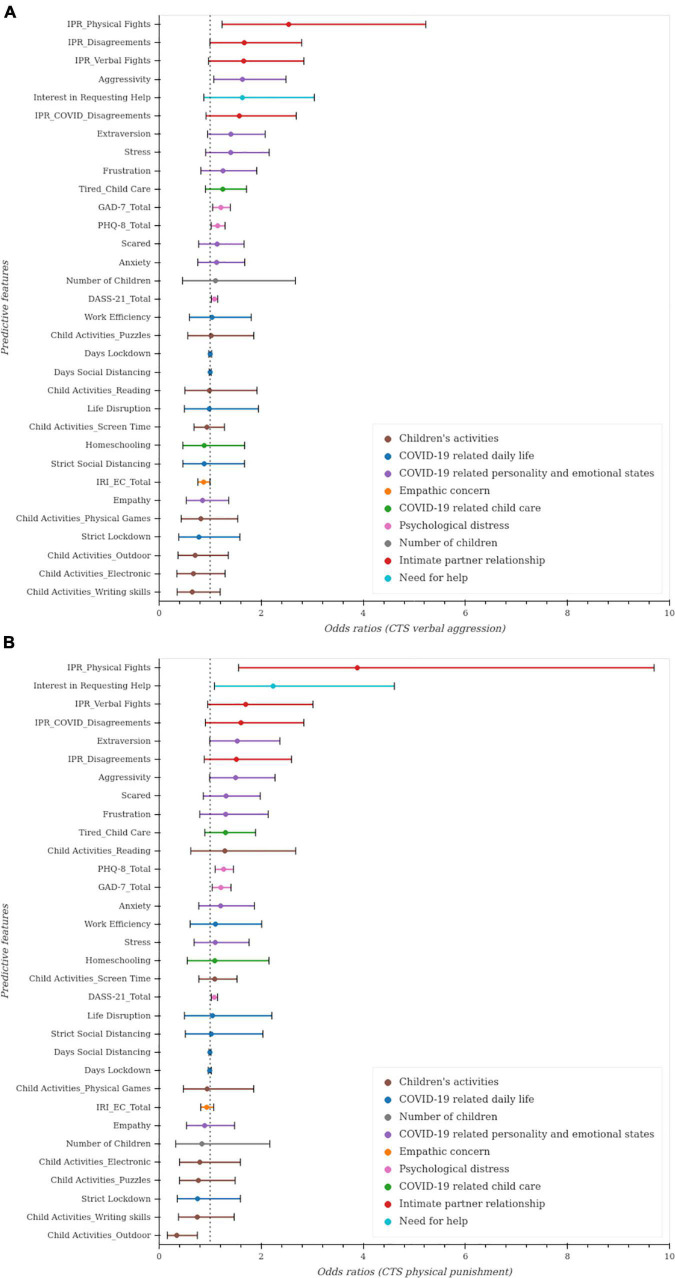
Unregularized regressions to predict indicators of child maltreatment. Unregularized logistic regression was used with 32 predictive factors after adjusting for age, gender and state region. Odds ratios and 99.84% confidence intervals (CI) for each predictor. The OR point estimates are represented by the dots and the line widths for each predictor present the 1- 0.05/32% = 99.84% CI adjusted for multiple comparisons using Bonferroni (as the gold standard method for multiple comparisons). Statistical significance is indicated by CI not including 1 at the 0.05/32 level. **(A)**. Predictors of verbal aggression toward children. **(B)**. Predictors of physical punishment toward children.

We modeled 32 variables as potential predictors and conducted an unregularized logistic regression to predict verbal aggression toward children while adjusting for age, gender, and state region. The odds ratios (OR) and 99.84% CI for each predictor are shown (Figure 1A; Table 2). Significant results were mainly observed for the *intimate partner relationship* category (physical fights, verbal fights, COVID-related disagreements, disagreements); for the *psychological distress* category (GAD-7, DASS-21, PHQ-8); for the *empathic concern* category (IRI_EC, empathy); for the *COVID-19 related personality and emotional states* category (aggressivity, extraversion, stress, frustration, anxiety, scared); for the *children’s activities* category (outdoor activities, physical games, electronic, writing skills); for the *COVID-19 related childcare* category (tired from childcare, homeschooling); and for *need for help* category.

**TABLE 2:** (Time 3): Predictors of verbal aggression toward children in the unregularized regression model.

Predictor variable	OR	*p*-value (adjusted)	99.8438% CI
IPR_Physical Fights	2.54	0.001	(1.23, 5.23)
IPR_Disagreements	1.67	0.055	(1.00, 2.79)
IPR_Verbal Fights	1.66	0.094	(0.97, 2.84)
Aggressivity	1.63	0.007	(1.07, 2.49)
Interest in Requesting Help	1.63	0.411	(0.88, 3.04)
IPR_COVID_Disagreements	1.57	0.250	(0.92, 2.69)
Extraversion	1.41	0.191	(0.95, 2.08)
Stress	1.40	0.414	(0.91, 2.16)
Frustration	1.25	1.000	(0.82, 1.91)
Tired_Child Care	1.25	0.872	(0.91, 1.71)
GAD-7_Total	1.21	≤0.001	(1.05, 1.40)
PHQ-8_Total	1.15	0.008	(1.02, 1.29)
Scared	1.14	1.000	(0.78, 1.67)
Anxiety	1.13	1.000	(0.76, 1.68)
Number of Children	1.10	1.000	(0.46, 2.67)
DASS-21_Total	1.08	≤0.001	(1.02, 1.15)
Work Efficiency	1.04	1.000	(0.60, 1.80)
Child Activities_Puzzles	1.02	1.000	(0.56, 1.85)
Days Lockdown	1.00	1.000	(0.97, 1.03)
Days Social Distancing	1.00	1.000	(0.98, 1.02)
Child Activities_Reading	0.99	1.000	(0.51, 1.92)
Life Disruption	0.98	1.000	(0.50, 1.95)
Child Activities_Screen Time	0.94	1.000	(0.69, 1.28)
Homeschooling	0.88	1.000	(0.47, 1.67)
Strict Social Distancing	0.88	1.000	(0.47, 1.67)
IRI_EC_Total	0.87	0.044	(0.76, 1.00)
Empathy	0.85	1.000	(0.53, 1.36)
Child Activities_Physical Games	0.82	1.000	(0.43, 1.54)
Strict Lockdown	0.78	1.000	(0.38, 1.58)
Child Activities_Outdoor	0.71	1.000	(0.37, 1.36)
Child Activities_Electronic	0.67	1.000	(0.35, 1.29)
Child Activities_Writing skills	0.65	0.806	(0.35, 1.20)

#### Physical punishment

We used similar methods to predict physical punishment (Figure 1B; Table 3). The greatest risk of physical punishment was associated with more physical fights, verbal fights, COVID-related disagreements, and disagreements between partners. Greater risk of physical punishment toward children was also associated with higher levels of anxiety and depression (PHQ-8, DASS-21, GAD-7), and with less empathic concern (IRI). COVID-19-related items to childcare (tired taking care of children), and to personality and emotional states (extraversion, aggressivity, frustration, scared, anxiety and stress) were also significant predictors. Children’s involvement in outdoor activities predicted less risk of physical punishment toward children.

**TABLE 3:** (Time 3): Predictors of physical punishment toward children in the unregularized regression model.

Predictor variable	OR	*p*-value (adjusted)	99.8438% CI
IPR_Physical Fights	3.88	≤0.001	(1.55, 9.70)
Interest in Requesting Help	2.23	0.014	(1.08, 4.61)
IPR_Verbal Fights	1.69	0.121	(0.95, 3.02)
IPR_COVID_Disagreements	1.60	0.285	(0.91, 2.84)
Extraversion	1.53	0.064	(0.99, 2.37)
IPR_Disagreements	1.51	0.472	(0.88, 2.59)
Aggressivity	1.50	0.067	(0.99, 2.27)
Scared	1.31	1.000	(0.87, 1.98)
Frustration	1.30	1.000	(0.80, 2.14)
Tired_Child Care	1.30	0.826	(0.90, 1.89)
Child Activities_Reading	1.29	1.000	(0.62, 2.68)
PHQ-8_Total	1.26	≤0.001	(1.10, 1.46)
GAD-7_Total	1.21	0.002	(1.04, 1.41)
Anxiety	1.20	1.000	(0.78, 1.87)
Work Efficiency	1.10	1.000	(0.61, 2.01)
Stress	1.10	1.000	(0.69, 1.76)
Homeschooling	1.09	1.000	(0.55, 2.15)
Child Activities_Screen Time	1.09	1.000	(0.78, 1.52)
DASS-21_Total	1.08	≤0.001	(1.02, 1.15)
Life Disruption	1.05	1.000	(0.50, 2.21)
Strict Social Distancing	1.02	1.000	(0.51, 2.03)
Days Social Distancing	1.00	1.000	(0.98, 1.02)
Days Lockdown	0.99	1.000	(0.96, 1.02)
Child Activities_Physical Games	0.94	1.000	(0.48, 1.85)
IRI_EC_Total	0.93	1.000	(0.81, 1.07)
Empathy	0.89	1.000	(0.54, 1.48)
Number of Children	0.84	1.000	(0.32, 2.17)
Child Activities_Electronic	0.80	1.000	(0.40, 1.59)
Child Activities_Puzzles	0.77	1.000	(0.40, 1.49)
Strict Lockdown	0.75	1.000	(0.36, 1.59)
Child Activities_Writing skills	0.75	1.000	(0.38, 1.47)
Child Activities_Outdoor	0.35	≤0.001	(0.16, 0.75)

### Regularized and machine learning models predicting child maltreatment

#### Verbal aggression and physical punishment

**FIGURE 2:**
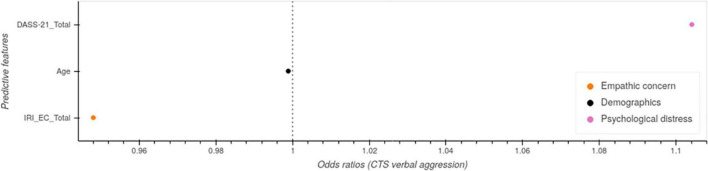
Regularized regression model predicting verbal aggression toward children (Time 3).

**FIGURE 3:**
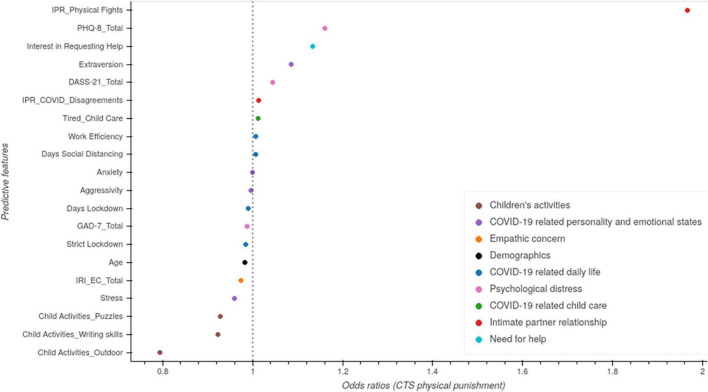
Regularized regression model predicting physical punishment toward children (Time 3).

We then estimated verbal aggression and physical punishment toward children using regularized logistic with elastic-net penalty, which allows us to account for many features while controlling for overfitting. For verbal aggression, we found empathic concern and DASS-21 scores best predicted verbal aggression (Figure 2). For physical punishment, we found that physical fights among partners, spending time outdoor, empathic concern and psychological distress are among the best predictive in the model (Figure 3). AUROC for cross validation was 0.88 ± 0.09 and 0.94 ± 0.05 with hyperparameters: *C* = 0.02, l1_ratio = 0.99 and *C* = 0.20, l1_ratio = 0.99, respectively.

**FIGURE 4:**
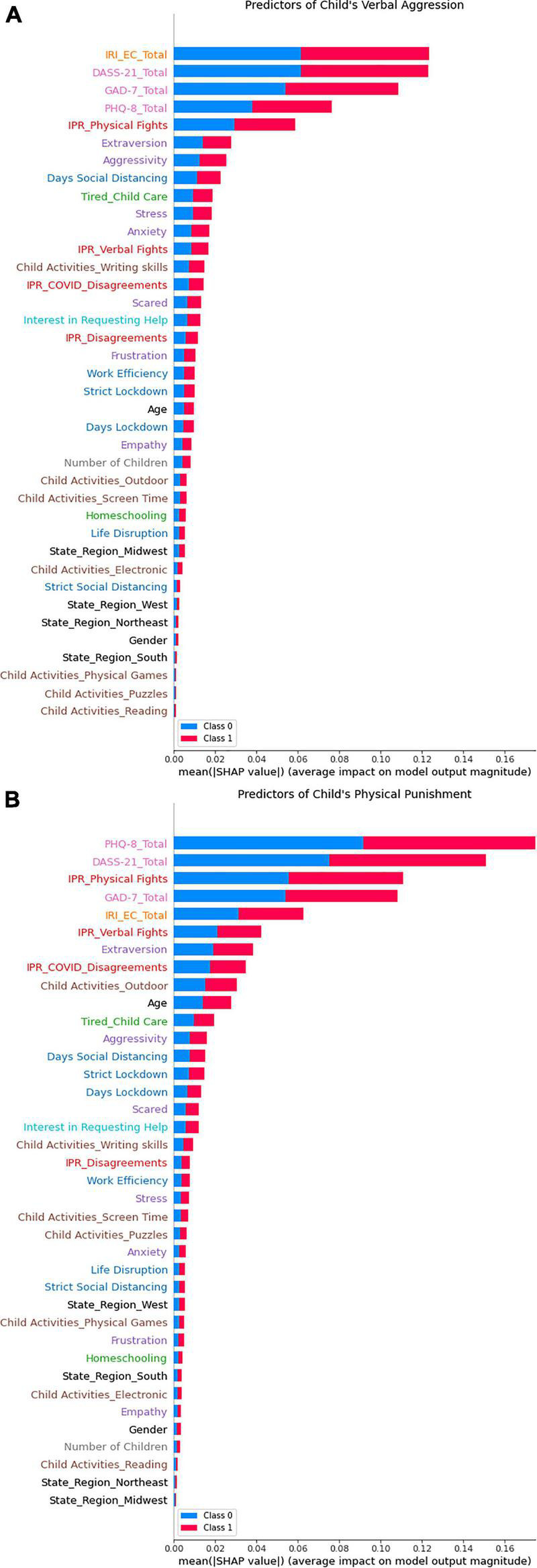
Random forest analysis to predict indicators of child maltreatment. Random forest was conducted with shapely additive explanations (SHAP) values. **(A)** Predictors of verbal aggression toward children. **(B)** Predictors of physical punishment toward children.

We then utilized a random forest classification model to determine if there were additional factors identified. For verbal aggression and physical punishment, empathic concern, psychological distress and physical fights among partners were the leading predictor of verbal aggression toward children (Figures 4A,B). Verbal fights among partners, COVID-related disagreements, extraverted personalities, and outdoor activities with children were among important factors that were associated with this physical punishment. The AUROC for the random forest was 0.91 ± 0.06 and 0.93 ± 0.06, with hyperparameters max_features = 0.36 and 0.22 and n_estimators = 211 and 446, respectively.

#### Predictors of intimate partner relationships

**FIGURE 5:**
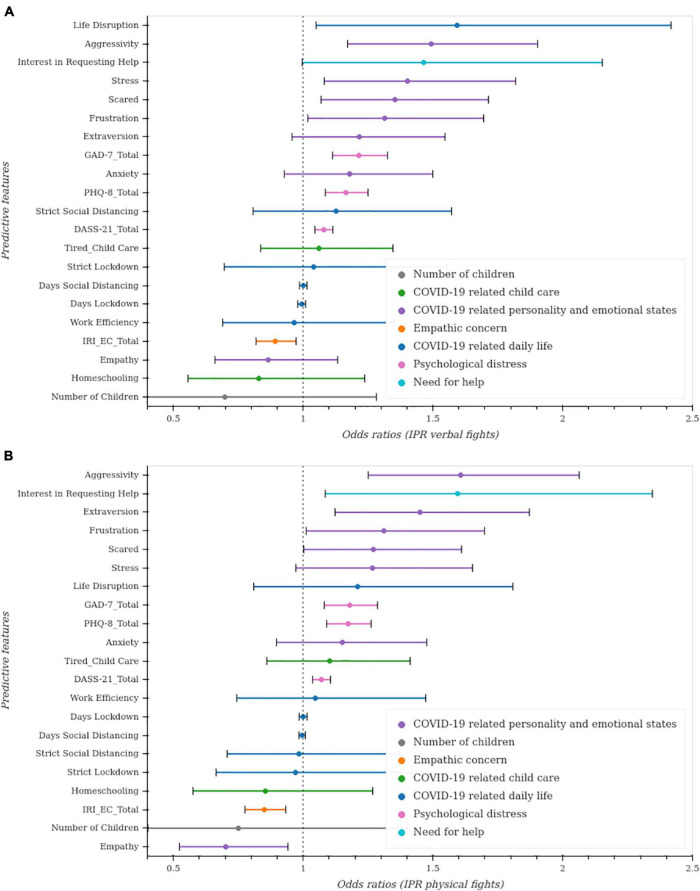
Unregularized regressions to predict intimate partner violence. Unregularized ordinal regressions were conducted. For each covariate of interest, an ordinal regression model was fit while controlling for age, gender, and state region. The *p*-values and CI for each model were then adjusted for multiple comparisons using the Bonferroni method. **(A)** Predictive factors of verbal fights between partners. **(B)** Predictive factors of physical fights between partners.

Unregularized regressions showed that COVID-19 related personality and emotional factors, psychological distress, and empathic concern are among the most predictors of verbal and physical fights (Figures 5A,B). Regularized regressions confirmed these results.

##### Verbal fights

We conducted both unregularized and regularized ordinal regression models to estimate verbal fights among partners (see Table 4). The unregularized model results demonstrate COVID-19 related personality and emotional factors (Figure 5A) (aggressivity, stressed, scared, frustration, extraversion), and interest in seeking help are among the most significant predictors of verbal fights among partners. Psychological distress (GAD-7, DASS-21, PHQ-8), and empathic concern (IRI_EC) are among the significant factors predicting an increase and a decrease in these verbal fights, respectively.

**TABLE 4:** (Time 3): Predictors of verbal fights between partners in the unregularized regression model.

Predictor variable	OR	*p*-value (adjusted)	99.7619% CI
Life Disruption	1.59	0.015	(1.05, 2.42)
Aggressivity	1.49	≤ 0.001	(1.17, 1.90)
Interest in Requesting Help	1.46	0.056	(1.00, 2.15)
Stress	1.40	0.002	(1.08, 1.82)
Scared	1.35	0.002	(1.07, 1.71)
Frustration	1.31	0.024	(1.02, 1.70)
Extraversion	1.22	0.275	(0.96, 1.55)
GAD-7_Total	1.21	≤0.001	(1.11, 1.33)
Anxiety	1.18	0.784	(0.93, 1.50)
PHQ-8_Total	1.16	≤0.001	(1.09, 1.25)
Strict Social Distancing	1.13	1.000	(0.81, 1.57)
DASS-21_Total	1.08	≤0.001	(1.05, 1.11)
Tired_Child Care	1.06	1.000	(0.84, 1.35)
Strict Lockdown	1.04	1.000	(0.70, 1.55)
Days Social Distancing	1.00	1.000	(0.99, 1.02)
Days Lockdown	0.99	1.000	(0.98, 1.01)
Work Efficiency	0.97	1.000	(0.69, 1.35)
IRI_EC_Total	0.89	0.001	(0.82, 0.97)
Empathy	0.87	1.000	(0.66, 1.13)
Homeschooling	0.83	1.000	(0.56, 1.24)
Number of Children	0.70	1.000	(0.38, 1.28)

**FIGURE 6:**
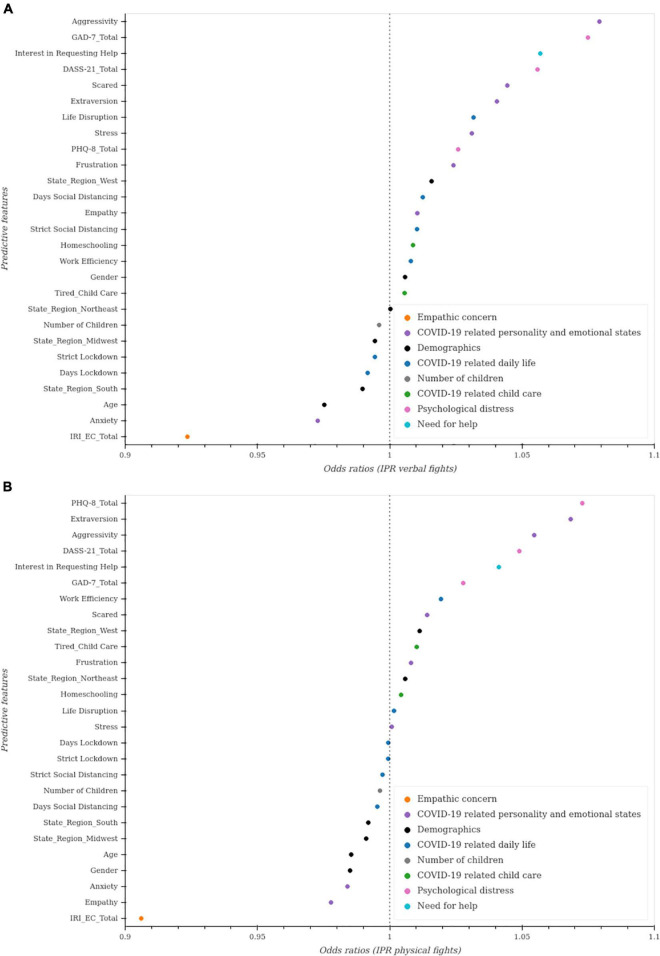
Regularized regression model predicting intimate partner verbal fights (Time 3) **(A)** and intimate partner physical fights (Time 3) **(B)**.

The regularized model used an ℓ_2_ penalty, the mean absolute error (MAE) 0.67=0.11 with a hyperparameter of α= 599.48. Note that this corresponds to the unit encoding of the Likert-scale response. The regularized model identified the same factors as predicting verbal fights (see Figure 6A).

##### Physical fights

We examined what factors were associated with increased physical fights among partners by conducting an ordinal regression (see Table 5). Of the factors included in the unregularized model (Figure 5B), the following were associated with increased physical fights among partners: interest in requesting help, COVID-19 related personality and emotional states (aggression, stress), anxious/worry, extraverted, frustrated, scared as well as psychological distress (GAD-7, DASS-21, PHQ-8). Empathic concern (IRI_EC) is predictive of less physical fights between partners. The MAE for the regularized model was 0.670.11 with a hyperparameter of α = 1204.50. Regularized regressions confirm the previous results (see Figure 6B).

**TABLE 5:** (Time 3): Predictors of physical fights between partners in the unregularized regression model.

Predictor variable	OR	*p*-value (adjusted)	99.7619% CI
Aggressivity	1.61	≤0.001	(1.25, 2.06)
Interest in Requesting Help	1.60	0.005	(1.09, 2.35)
Extraversion	1.45	≤0.001	(1.12, 1.87)
Frustration	1.31	0.031	(1.01, 1.70)
Scared	1.27	0.045	(1.00, 1.61)
Stress	1.27	0.142	(0.97, 1.65)
Life Disruption	1.21	1.000	(0.81, 1.81)
GAD-7_Total	1.18	≤0.001	(1.08, 1.29)
PHQ-8_Total	1.17	≤0.001	(1.09, 1.26)
Anxiety	1.15	1.000	(0.90, 1.48)
Tired_Child Care	1.10	1.000	(0.86, 1.41)
DASS-21_Total	1.07	≤0.001	(1.04, 1.11)
Work Efficiency	1.05	1.000	(0.74, 1.47)
Days Lockdown	1.00	1.000	(0.98, 1.02)
Days Social Distancing	1.00	1.000	(0.98, 1.01)
Strict Social Distancing	0.98	1.000	(0.71, 1.37)
Strict Lockdown	0.97	1.000	(0.66, 1.42)
Homeschooling	0.85	1.000	(0.58, 1.27)
IRI_EC_Total	0.85	≤0.001	(0.78, 0.93)
Number of Children	0.75	1.000	(0.40, 1.40)
Empathy	0.70	0.005	(0.52, 0.94)

### Aim 2: Increase in family violence at time 1, time 2, and time 3

At Times 1 and 2 on average, 58.63% reported one or more instances of verbal aggression toward children, 22.39% reported one or more instance of physical punishment toward children, 21.46% reported agreeing or strongly agreeing that they had more verbal fights with their partner than usual, and 1.32% reported agreeing or strongly agreeing that they had more physical fights with their partner than usual (more information about each time point in Table 6). We conducted logistic regression for verbal and physical punishment toward children and found a significant increase for physical punishment between Times 1, 2, and 3. There is an increased risk for physical punishment that was associated with time (OR = 4.32; CI = 2.65, 7.02). While there was no effect of time for verbal aggression, there was a significant interaction between male gender and time, signifying an increased risk for verbal aggression over time only in males (OR = 2.4, 2; CI = 1.24, 3.95). Ordinal regressions also revealed a significant increase risk of verbal and physical fights between partners (OR = 2.19, CI = 1.6, 2.99; OR = 5.71, CI = 3.86, 8.45, respectively).

**TABLE 6:** Descriptive data for Times 1 and 2.

A									
		**CTS_Physical_Punishment**							
		
	**Time**	**Time 1**				**Time 2**			
			
		**Count**	**Percent**	**chi2**	***p*-value**	**Count**	**Percent**	**chi2**	***p*-value**
		
**Features**	**Value**								

Race	American Indian/Alaskan Native	2	0.71%	14.30982	0.02636	2	0.52%	9.409253	0.151836
	Asian/Native Hawaiin/Other Pacific Islander	9	3.21%	14.30982	0.02636	7	1.81%	9.409253	0.151836
	Biracial	4	1.43%	14.30982	0.02636	17	4.40%	9.409253	0.151836
	Black/African American/African Diaspora	32	11.43%	14.30982	0.02636	46	11.92%	9.409253	0.151836
	Hispanic	25	8.93%	14.30982	0.02636	38	9.84%	9.409253	0.151836
	Other	2	0.71%	14.30982	0.02636	4	1.04%	9.409253	0.151836
	White/Caucasian	206	73.57%	14.30982	0.02636	272	70.47%	9.409253	0.151836
Gender	Female	163	58.21%	1.542525	0.214242	258	66.84%	0.60019	0.438506
	Male	117	41.79%	1.542525	0.214242	128	33.16%	0.60019	0.438506
State_Region	Midwest	61	21.94%	2.311449	0.510331	80	20.89%	13.17189	0.004279
	Northeast	38	13.67%	2.311449	0.510331	49	12.79%	13.17189	0.004279
	South	123	44.24%	2.311449	0.510331	182	47.52%	13.17189	0.004279
	West	56	20.14%	2.311449	0.510331	72	18.80%	13.17189	0.004279
Income	$10−20k	24	8.57%	13.06126	0.042073	66	17.05%	14.54931	0.024067
	$20−30k	24	8.57%	13.06126	0.042073	59	15.25%	14.54931	0.024067
	$30−40k	27	9.64%	13.06126	0.042073	61	15.76%	14.54931	0.024067
	$40−50k	34	12.14%	13.06126	0.042073	45	11.63%	14.54931	0.024067
	$50−70k	51	18.21%	13.06126	0.042073	48	12.40%	14.54931	0.024067
	$70−90k	52	18.57%	13.06126	0.042073	46	11.89%	14.54931	0.024067
	$90k or more	68	24.29%	13.06126	0.042073	62	16.02%	14.54931	0.024067
Education	some high school	1	0.36%	6.168354	0.29018	6	1.55%	2.659543	0.752299
	high school grad	27	9.61%	6.168354	0.29018	47	12.11%	2.659543	0.752299
	some college	69	24.56%	6.168354	0.29018	120	30.93%	2.659543	0.752299
	associate’s degree	34	12.10%	6.168354	0.29018	47	12.11%	2.659543	0.752299
	bachelor’s degree	105	37.37%	6.168354	0.29018	123	31.70%	2.659543	0.752299
	master’s, professional, doctorate	45	16.01%	6.168354	0.29018	45	11.60%	2.659543	0.752299
CTS_Physical_Punishment	No physical punishment	225	80.07%			305	77.61%		
	One or more instances of physical punishment	56	19.93%			88	22.39%		

B									

		**CTS_Physical_Punishment**							
		
	**Time**	**Time 1**				**Time 2**			
			
		**Count**	**Percent**	**chi2**	***p*-value**	**Count**	**Percent**	**chi2**	***p*-value**
		
**Features**	**Value**								

Race	American Indian/Alaskan Native	2	0.71%	8.389159	0.210956	2	0.52%	5.326317	0.502692
	Asian/Native Hawaiin/Other Pacific Islander	10	3.55%	8.389159	0.210956	7	1.81%	5.326317	0.502692
	Biracial	4	1.42%	8.389159	0.210956	17	4.39%	5.326317	0.502692
	Black/African American/African Diaspora	32	11.35%	8.389159	0.210956	46	11.89%	5.326317	0.502692
	Hispanic	25	8.87%	8.389159	0.210956	38	9.82%	5.326317	0.502692
	Other	2	0.71%	8.389159	0.210956	4	1.03%	5.326317	0.502692
	White/Caucasian	207	73.40%	8.389159	0.210956	273	70.54%	5.326317	0.502692
Gender	Female	164	58.16%	5.195261	0.022649	258	66.67%	0.585278	0.444251
	Male	118	41.84%	5.195261	0.022649	129	33.33%	0.585278	0.444251
State_Region	Midwest	61	21.79%	1.935782	0.585842	81	21.09%	0.783118	0.853501
	Northeast	40	14.29%	1.935782	0.585842	49	12.76%	0.783118	0.853501
	South	123	43.93%	1.935782	0.585842	181	47.14%	0.783118	0.853501
	West	56	20.00%	1.935782	0.585842	73	19.01%	0.783118	0.853501
Income	$10−20k	24	8.51%	4.581145	0.59854	66	17.01%	5.467596	0.485382
	$20−30k	24	8.51%	4.581145	0.59854	58	14.95%	5.467596	0.485382
	$30−40k	27	9.57%	4.581145	0.59854	62	15.98%	5.467596	0.485382
	$40−50k	34	12.06%	4.581145	0.59854	45	11.60%	5.467596	0.485382
	$50−70k	53	18.79%	4.581145	0.59854	48	12.37%	5.467596	0.485382
	$70−90k	52	18.44%	4.581145	0.59854	47	12.11%	5.467596	0.485382
	$90k or more	68	24.11%	4.581145	0.59854	62	15.98%	5.467596	0.485382
Education	some high school	1	0.35%	3.142705	0.677996	6	1.54%	2.578972	0.764558
	high school grad	27	9.54%	3.142705	0.677996	47	12.08%	2.578972	0.764558
	some college	69	24.38%	3.142705	0.677996	121	31.11%	2.578972	0.764558
	associate’s degree	35	12.37%	3.142705	0.677996	46	11.83%	2.578972	0.764558
	bachelor’s degree	105	37.10%	3.142705	0.677996	123	31.62%	2.578972	0.764558
	master’s, professional, doctorate	46	16.25%	3.142705	0.677996	46	11.83%	2.578972	0.764558
CTS_Physical_Punishment	No physical punishment								
	One or more instances of physical punishment								
CTS_Verbal_Agression_2items	No verbal abuse	107	37.81%			163	41.37%		
	One or more instances of verbal abuse	176	62.19%			231	58.63%		

Logistic regression showed that there is a significant increased risk of physical punishment for certain states [West (OR = 2.81, CI = 1.62, 4.88), South (OR = 2.23, CI = 1.36, 3.68), and Northeast (OR = 2.14, CI = 1.13, 4.04)] as compared to Midwest. Also, larger incomes were associated with less risk of physical punishment (OR = 0.33, CI = 0.17, 0.63). In terms of physical fights between partners, there was a significant increase of risk in the West as compared to Midwest (OR = 2.66, CI = 1.71, 4.14). There was also an increased risk for male reporting on physical fights as compared to females (OR = 1.68, CI = 1.25, 2.27). For physical punishment, verbal aggression toward children and physical fights between partners, there was a significant effect of time (OR = 2.32, CI = 1.55, 3.47; OR = 1.49, CI = 1.06, 2.1; OR = 2.78, CI = 1.96, 3.94), which means that there was a lager increase between Time 3 and Time 2 than Time 2 compared to Time 1.

## Discussion

We used machine learning analysis to assess the strongest predictors of indicators of child maltreatment and intimate partner violence. We found empathic concern and depression and anxiety symptoms were among the strongest predictors of verbal aggression toward children. Physical fights among parents as well as specific traits and behaviors (such as being extraverted and aggressive) were also among the important factors identified in the machine learning model. However, for physical punishment toward children, a greater number of factors related to parents’ relationship such as physical fights, verbal fights, and COVID-19 related disagreements were strongly associated with this form of maltreatment. Indicators of anxiety, depression, lower empathy, and extraversion were among the strongest predictors of increased physical punishment toward children. Finally, children’s outdoor activities were among the strongest predictors of less physical punishment.

Interestingly, for both verbal aggression and physical punishment, parental psychological distress and children’s activities factors were ranked as significantly more important than elements related to strict social distancing or lockdown. However, several other factors that are directly related to the pandemic, such as parents’ disagreements related to COVID-19, being tired from taking care of children during COVID-19, and emotional states during the pandemic are significant contributors to child maltreatment. Research demonstrates past pandemics have had a negative impact on psychological well-being and mental health. During the 2003 SARS outbreak, a 31.2% increase in depression and 28.9% increase in anxiety (21). Recent studies have found increased psychological difficulties during the crisis (22). Psychological difficulties in caregivers are associated with increased risk for child maltreatment (23). Our findings corroborate these studies and show that increased anxiety and depression in parents are correlated with greater risk for child maltreatment. When considered together, results of this study illustrate a concerning picture of increased parental distress relates to increased aggression toward children. Parents’ psychological distress was an even more important predictor than the number of days during lockdown. Therefore, providing support to parents may prevent maltreatment.

Additionally, since lack of empathy is considered one of the roots of aggression (24), empathy training has been used in parenting intervention programs for child maltreatment (25). Abusive fathers were less affectively and cognitively empathic toward their children (26). Other studies found less parental affective but not cognitive empathy was associated with higher risk of child physical abuse (27). The current study suggests empathic concern as well as emotion regulation and non-conflictual relationships between parents may be critical objectives in efforts to prevent child maltreatment.

Our findings demonstrate physical fights among partners and symptoms of depression were the leading predictors of physical punishment and verbal aggression toward children. Isolation poses serious risks for the perpetration of IPV. Factor characteristics of IPV, such as social isolation from supportive peers, functional isolation from support services, and excessive monitoring and control of victims’ activities, are also results of the pandemic and its associated orders (28). Our findings show empathic concern was among the best predictor of lower risk of violence among partners.

Finally, we assessed the effect of time on family violence and found that physical punishment toward children as well as partner violence increased later in the pandemic as compared to the beginning of the pandemic. Given that COVID-19 pandemic is still alarming today, we believe increasing awareness about family violence is crucial.

Even though we are testing here as proxy indicators of child maltreatment, these indicators are not as distinct from physical maltreatment, but instead related to a continuum form of aggressive acts toward children that can impact child’s development (29). We also used yelling/screaming at children as a proxy indicator of emotional abuse that has a negative impact on a child’s emotional health (30).

Our study had several limitations. We used a self-report measurement to acquire predictors of child maltreatment and IPV. Our study is therefore examining indicators of abuse. Our sample is also limited to Amazon’s Mechanical Turk and Prolific. Additionally, for the comparison in child maltreatment between different times of the pandemic, given that we used data of independent samples, there might be other confounders not accounted for that may have impacted the rates of child abuse. We have accounted for different factors such as education and income. However there might be a potential complex dependence structure between these independent factors as they can be correlated.

Given the markedly high rates of both IPV and indicators of child maltreatment compared to the previous years (31), it is critical that educators and professionals actively screen for indicators of abuse. Parenting interventions such as the ACT Parents Raising Safe Kids program (32) aim to improve parental nurturing and emotion regulation. It is also imperative that protective mechanisms are put in place to safeguard children from maltreatment during the pandemic and beyond. Increased efforts to educate healthcare, mental health care, social service providers and other professionals on recognizing and responding to child maltreatment through both in-person and virtual service provision also will be necessary (33). Programs such as the Child Advocacy Studies Training program (CAST) that educate undergraduate and graduate students in a wide variety of fields about child maltreatment identification and responding may effectively begin to address this goal (34). Policy-makers, child welfare and family-serving organizations should prioritize the development of safe and confidential spaces for children to discuss their concerns and experiences; some nations have developed models for this (33). Screening for child maltreatment can be broadened and placed in a variety of institutions including childcare centers, health clinics, and faith-based organizations (35). Innovative methods to reach children during critical times should be identified. For example, social media and virtual platforms are being explored as potential tools for improved understanding and monitoring of children’s experiences with family violence as well as improved accurate reporting of child maltreatment (36, 37). For youths traumatized by child maltreatment, trauma-informed telemental health services and remote case management are critical (8, 37). Lastly, programs addressing parenting practices and parenting stress should be destigmatized, and made to be universally available, including being readily accessible online (8, 35, 37).

## Data availability statement

The raw data supporting the conclusions of this article will be made available by the authors, without undue reservation.

## Ethics statement

The studies involving human participants were reviewed and approved by Human Research Protection Program at the University of Toledo. The patients/participants provided their written informed consent to participate in this study.

## Author contributions

KT designed research, collected data, performed analysis and interpretation, and co-wrote the manuscript. EO’L performed analysis and co-write the manuscript. KW designed study, collected data, and performed analysis. PD collected data and performed analysis. SL designed research, collected data, performed analysis and interpretation. MK designed research, performed analysis and interpretation, and co-wrote the manuscript. EA conceived and designed research, performed data analysis and interpretation, and co-wrote the manuscript. All authors contributed to the article and approved the submitted version.
